# Mannosylated-serum albumin nanoparticle imaging to monitor tumor-associated macrophages under anti-PD1 treatment

**DOI:** 10.1186/s12951-023-01791-9

**Published:** 2023-01-27

**Authors:** Gyo Jeong Gu, Hyewon Chung, Ji Yong Park, Ranji Yoo, Hyung-Jun Im, Hongyoon Choi, Yun-Sang Lee, Seung Hyeok Seok

**Affiliations:** 1 Macrophage Laboratory, Department of Microbiology and Immunology, Institute of Endemic Disease, Seoul National University College of Medicine, Seoul, Republic of Korea; 2 Department of Nuclear Medicine, Seoul National University College of Medicine, Seoul, Republic of Korea; 3 Department of Molecular Medicine and Biopharmaceutical Sciences, Graduate School of Convergence Science and Technology, Seoul National University, Seoul, Republic of Korea; 4 Dental Research Institute, Seoul National University, Seoul, Republic of Korea; 5 Radiation Research Institute, Seoul National University College of Medicine, Seoul, Republic of Korea; 6 Cancer Research Institute, Seoul National University College of Medicine, Seoul, Republic of Korea; 7 Department of Biomedical Sciences, Seoul National University College of Medicine, Seoul, Republic of Korea; 8 Department of Nuclear Medicine, Seoul National University Hospital, Seoul, Republic of Korea; 9 Biomedical Research Institute, Seoul National University Hospital, Seoul, Republic of Korea; 10 Bio-MAX Institute, Seoul National University, Seoul, Republic of Korea

**Keywords:** Mannosylated-serum albumin, Positron emission tomography, Tumor microenvironment, Tumor-associated macrophage, Anti-PD1

## Abstract

**Background:**

Immune checkpoint inhibitors such as anti-programmed cell death protein 1 (PD1) block tumor growth by reinvigorating the immune system; however, determining their efficacy only by the changes in tumor size may prove inaccurate. As the immune cells including macrophages in the tumor microenvironment (TME) are associated with the response to anti-PD1 therapy, tumor-associated macrophages (TAMs) imaging using nanoparticles can noninvasively provide the immune enrichment status of TME. Herein, the mannosylated-serum albumin (MSA) nanoparticle was labeled with radioactive isotope ^68^Ga to target the mannose receptors on macrophages for noninvasive monitoring of the TME according to anti-PD1 therapy.

**Results:**

B16F10-Luc and MC38-Luc tumor-bearing mice were treated with anti-PD1, and the response to anti-PD1 was determined by the tumor volume. According to the flow cytometry, the responders to anti-PD1 showed an increased proportion of TAMs, as well as lymphocytes, and the most enriched immune cell population in the TME was also TAMs. For noninvasive imaging of TAMs as a surrogate of immune cell augmentation in the TME via anti-PD1, we acquired [^68^Ga] Ga-MSA positron emission tomography. According to the imaging study, an increased number of TAMs in responders at the early phase of anti-PD1 treatment was observed in both B16F10-Luc and MC38-Luc tumor-bearing mice models.

**Conclusion:**

As representative immune cells in the TME, non-invasive imaging of TAMs using MSA nanoparticles can reflect the immune cell enrichment status in the TME closely associated with the response to anti-PD1. As non-invasive imaging using MSA nanoparticles, this approach shows a potential to monitor and evaluate anti-tumor response to immune checkpoint inhibitors.

**Supplementary Information:**

The online version contains supplementary material available at 10.1186/s12951-023-01791-9.

## Background

Antibodies against programmed cell death protein 1 (PD1) or programmed death-ligand 1 (PD-L1) are immune checkpoint inhibitors (ICIs) that exert their anti-tumor effects by reinvigorating T cells in the tumor microenvironment (TME) [1]. Although treatments with anti-PD1 can induce sustained responses by awakening the immune system, tumor progression occurs in several patients after treatment [2]. Therefore, early response evaluation to determine the continuation of ICIs is an essential aspect of therapeutic strategy design. However, response evaluation for ICIs is difficult because of pseudo-progression and hyper-progression, which limits early response evaluation using conventional imaging methods [3, 4]. Therefore, determining the therapeutic effects of ICIs using mechanism-based biomarkers such as non-invasive visualization of immune cells is highly desirable.

Most studies regarding responsiveness to anti-PD1 therapy using Immuno-PET imaging have focused on CD8^+^ T cells and NK cells, which can kill tumor cells directly [5–8]. These studies examined numerical increases or changes in the distribution of T cells in tumors to interpret the therapeutic response to treatment in patients and confirmed the levels of T cell-secreted cytokines IFNɣ and Granzyme B as non-invasive indicators of immune response activation [9–13]. However, not only the amount of labels at the site has appeared disparate from the actual cell numbers since the cell expansion and loss of cytokine activity, but there are also problems with the specific affinity to target, difficulties in conjugation, and in vivo tracer stability, as well as advanced technology to produce pure mAbs commercially [14].

Tumor-associated macrophages (TAMs) are the most abundant immune cells of the tumor microenvironment in solid tumors. They display remarkable heterogeneity and plasticity depending on the TME and are highly correlated with therapeutic efficacy [15–20]. However, non-invasive monitoring of TAMs after therapy has not been investigated, despite their ability to modulate T cell activation and control immune responses through the production of various cytokines [21–24]. Therefore, longitudinally monitoring the changes in TAMs during anti-PD1 therapy could expand our understanding of the therapeutic effects of ICIs and yield a more accurate prediction of the therapy response.

In this study, we used ^68^Ga-labeled mannosylated- serum albumin (MSA) nanoparticle to trace TAMs after anti-PD1 treatment non-invasively. MSA nanoparticle was developed as a macrophage-specific tracer by binding to a mannose receptor (CD206) expressed by macrophage [25]. In previous studies, [^68^Ga]Ga-MSA PET imaging can help diagnose pulmonary artery hypertension and monitor the inflammatory status by imaging the degree of mannose receptor-positive macrophage infiltration into the lung [26]. In addition, [^68^Ga]Ga-MSA PET imaging also visualizes the infiltration of mannose receptor-positive macrophages in monitoring myocarditis's treatment response [27]. Through these studies, we assumed that the [^68^Ga]Ga-MSA PET imaging could be a novel noninvasive diagnostic and monitoring tool for mannose receptor-positive TAM after immunotherapy.

**Fig. 1:**
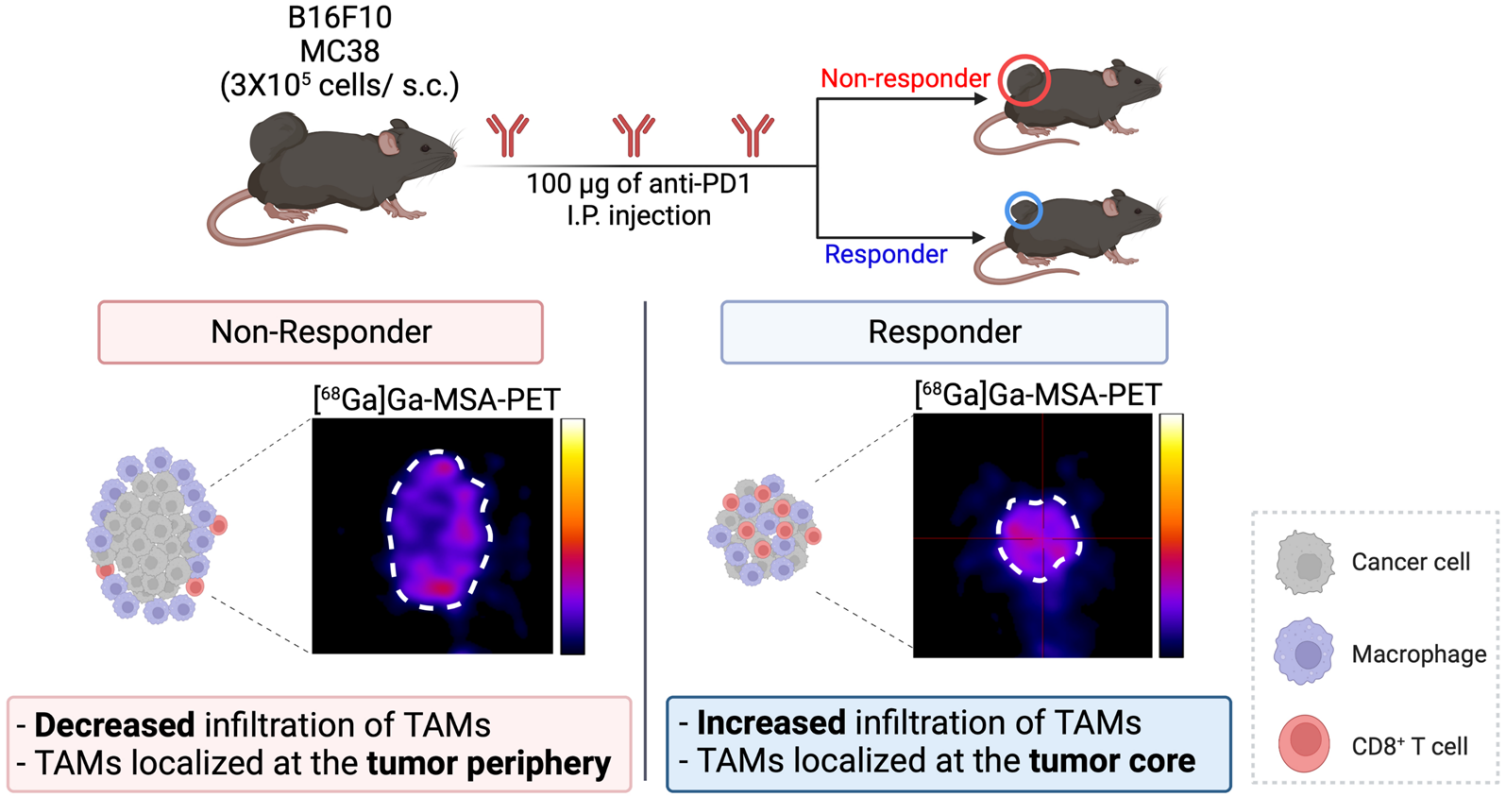
Schematic illustration of the [^68^Ga]Ga-MSA nanoparticle imaging in monitoring tumor-associated macrophages of tumor microenvironment

Based on the optimized MSA nanoparticle imaging, our results showed that the TAMs moved into the core of the tumor in responders and that the number of TAMs increased significantly (Fig. 1). Through these results, we suggest that non-invasive monitoring TAMs using MSA nanoparticle imaging can help monitor and predict the response to ICI therapy.

## Materials and methods

### Mice and tumor models

7–10 weeks-old wild-type (WT) C57BL/6 female mice were used in all the experiments. B16F10-luciferase expressing (B16F10-Luc) melanoma cells and MC38-luciferase expressing (MC38-Luc) colon adenocarcinoma cells were used in all experiments. B16F10-Luc cells were cultured in vitro in RPMI-1640 with L-glutamine medium supplemented with 10% fetal bovine serum (FBS) and 1% penicillin/streptomycin (PS) at 37 °C in a humidified incubator containing 5% CO_2_. MC38-Luc cells were cultured in vitro in DMEM medium supplemented with 10% FBS and 1% PS at 37 °C in a humidified incubator containing 5% CO_2_. Tumor volume was calculated using the formula (width^2^ × length)/2 every 3–4 d. At 7 or 10 d after tumor implantation, 100 μg anti-PD1 (clone 29F.1A12, BioXcell, USA) was administered three times via intraperitoneal injection. Animals that did not grow tumors or had tumors smaller than 5 mm in diameter on the 1 day of anti-PD1 treatment were excluded. After the last imaging, the tumors were collected for histology, flow cytometry, and PCR analysis.

### Immunohistochemistry

Immunohistochemistry was performed according to the manufacturer’s instructions. Briefly, paraffin-embedded sections were deparaffinized, hydrated, and antigen retrieved by steaming the slides in sodium citrate buffer (10 mM sodium citrate, pH 6.0). The slides were then treated with 3% H_2_O_2_ and blocked using a blocking solution. Primary antibodies were pre-diluted in blocking solution at 1:200 for CD206 (R&D Systems) and applied to tissue sections overnight at 4 °C in a humidified chamber. Biotinylated secondary antibodies were then applied, followed by signal development using liquid DAB substrate (Dako). Sections were counterstained with hematoxylin (Abcam). The slides were scanned using a digital camera Aperio AT2 (Leica). Analysis of immunohistochemistry staining intensity was performed using ImageJ through the IHC profiler plugin.

### Flow cytometry

The s.c. tumors were minced and then digested in HBSS—10% FBS containing 2 mg/ml collagenase type I (Sigma Aldrich) and 10 μg/ml DNase I (Sigma Aldrich) at 37 °C for 30 min to digestion. Digested cells were passed through a 70 μm pore-size cell strainer to prepare single cell suspensions for flow cytometry. Anti-mouse CD16/32 antibody (93, BioLegend) was pre-added to block the non-specific binding of the immunoglobulin to macrophage Fc receptors. For surface marker analysis, live cells were re-suspended in FACS buffer (1% BSA, 0.1% sodium azide in PBS) and stained with anti-mouse CD45 (30-F11), F4/80 (BM8), Ly6C (HK1.4), CD11b (M1/70, eBioscience), Ly6G (RB6-8C5) (BioLegend), CD206 (MR6F3), CD3 (145-2C11), CD8α (53–6.7), and CD4 (RM4-5) (eBioscience) at 4 °C for 20 min. For intracellular staining, cells were incubated for 2 h with GolgiPlug (BD Biosciences) and GolgiSTOP (BD Biosciences) at 1 μl/ml of culture media. Cells were then surface stained and then fixed and permeabilized using Foxp3/Transcription factor staining Buffer (Invitrogen), labeled with Granzyme B (NGZB, eBioscience) and IFN-γ (XMG1.2, BD Biosciences). Data were acquired using an LSRFortessa system (BD Biosciences) and analyzed with FlowJo software version 10.8.1. (BD Biosciences).

### Quantitative real-time PCR

Total RNA was extracted from the tumor, and tumor explant supernatant (TES) educated macrophages using TRIzol reagent (Invitrogen) according to the manufacturer’s instructions. cDNA was reverse transcribed from 1 mg of total RNA, and the amount of mRNA was determined by real-time PCR using the SYBR Green qPCR Pre-Mix (Enzynomics). All samples were normalized to the 18S rRNA mRNA expression levels. The primer sequences are listed in Additional file 3: Table S1.

### Isolation of primary macrophage and preparation of tumor explant supernatant (TES)

Primary macrophages were differentiated from mouse bone marrow cells. Bone marrow cells were obtained from 7–10 week-old C57BL/6 male mice and differentiated into mature bone marrow-derived macrophages (BMDMs) for 7 d in RPMI 1640 containing 10% FBS, 1% PS, and 2 mM L-glutamine (GIBCO), supplemented with fresh recombinant murine macrophage-colony stimulating factor (M-CSF) (50 ng/ml; Miltenyi Biotec). The medium was replaced on days 3 and 5 with a fresh medium containing M-CSF. For preparing TES, tumors from B16F10-Luc-tumor bearing mice were dissected and minced using a blade in a cell culture plate. After chopping, tumors were incubated in a humidified incubator at 37 °C with 5% CO_2_ with RPMI media (1 g tumor/15 mL RPMI media). Supernatants were collected after 24 h. To remove the debris, the supernatant was centrifuged at 12,000 ×*g*, 4 °C and filtered using a 0.2 μm pore size syringe filter.

### Synthesis of [.^68^ Ga] Ga-mannosylated- serum albumin (MSA)

For synthesizing [^68^Ga]Ga-MSA using click chemistry, we used a modified method described in our previous report [28, 29]. Briefly, albumin (50 mg, 757 nmol) in phosphate-buffered saline (PBS, 0.5 mL, pH 7.4) was reacted with azadibenzocyclooctyne-PEG_4_-*N*-hydroxysuccinimidyl ester (ADIBO-NHS, 4 mg, 8.4 μmol) in DMSO (10 µL). The reaction mixture was kept at 4 °C for 4 h and purified using a centrifugal filter unit (30 kDa) to form ADIBO-albumin (42 mg, 6.05 nmol). The number of ADIBO groups on albumin was determined to be 9.3 by matrix-assisted laser desorption/ionization-time of flight (MALDI-TOF). Mannosyl groups on albumin were introduced by a click reaction with ADIBO-albumin and azido-mannose (Man-N_3_). Man-N_3_ (1.6 mg, 4.8 μmol) in PBS (100 µL) was added to ADIBO-albumin (42 mg, 6.05 nmol) in PBS (100 µL), and the reaction mixture was kept at 4 °C for 2 h and purified using a desalting column (PD-10) with PBS as an eluent to yield mannosylated- serum albumin (MSA, 39.9 mg, 588 nmol). The number of mannosyl groups on albumin was determined using MALDI-TOF to be 6.6. The size of ADIBO-albumin and MSA was measured as 7.101 ± 1.021 nm and 8.534 ± 1.224 nm, respectively, using dynamic light scattering. MSA was then labeled with fluorescent dyes (FL) and radioisotopes in the residual ADIBO groups. For fluorescence labeling, MSA (588 nmol/0.5 mL PBS) was reacted with FNR648-N_3_ (44 μg, 58 nmol) in DMSO (6 µL) for 30 min. The reaction mixture was purified using a centrifugal filter (30 kDa) and concentrated to yield fluorescence-labeled MSA (MSA-FL, 500 nmol/0.5 mL in PBS). Lastly, MSA-FL was radiolabeled with gallium-68 (^68^Ga, half-life: 68 min). For radiolabeling, 3-azidopropyl-NOTA (NOTA-N_3_, 10 μg, 18 nmol) in 0.1 M sodium acetate buffer (pH 5.5, 1 mL) was mixed with freshly eluted ^68^ Ga (370 mBq) in 0.1 M HCl solution (1 mL) to give [^68^Ga]Ga-NOTA-N_3_ with quantitative yield. MSA-FL (7.1 mg, 100 nmol) was then reacted with [^68^Ga]Ga-NOTA-N_3_ (37 MBq/200 μL) to yield radiolabeled MSA-FL ([^68^Ga]Ga-MSA) with quantitative yield. After radiolabeling, [^68^Ga]Ga-MSA was used for PET imaging without further purification. The radiochemical purity of [^68^Ga]Ga-MSA was > 99%, and the *R*_f_ values of the free ^68^ Ga, [^68^Ga]Ga-NOTA-N_3_, and [^68^Ga]Ga-MSA were 0.9–1.0, 0.7–0.8, and 0.0–0.1, respectively, in radio-TLC.

### PET image acquisition and analysis

A small animal PET system (Genisys PET box) was used for PET acquisition for 5 min 2 h after intravenous injection of [^68^Ga]Ga-MSA. The activity of the injected [^68^Ga]Ga-MSA was 50 μCi/0.1 mL. Reconstruction of the PET images was automatically performed using vendor-provided software. The [^68^Ga]Ga-MSA of each tumor was assessed by a semi-quantitative method using LifeX software version 7.0.15. The maximum counts of tumors were measured using manual volumes-of-interest placed on the tumor lesions. For count normalization, another manual spherical volume-of-interest was placed on the mediastinum to reflect blood pool counts. To quantify [^68^Ga]Ga-MSA uptake, we used LBP which is counts for MSA uptake in the tumor normalized by counts of blood pool. This approach is a semi-quantitative method for PET in oncology for response and inter-subject comparison to reduce variability due to technical factors [30].

### Statistical analysis

All statistical analyses were conducted using GraphPad Prism software (version 8.0) and are displayed as the mean ± S.E.M. Statistical significance was assessed using Student’s t-test, and ANOVA with Tukey’s post-test was performed for multiple comparisons.

## Results

### The effective anti-PD1 treatment induces increased frequency and activity of CD8^+^ T cells

**Fig. 2:**
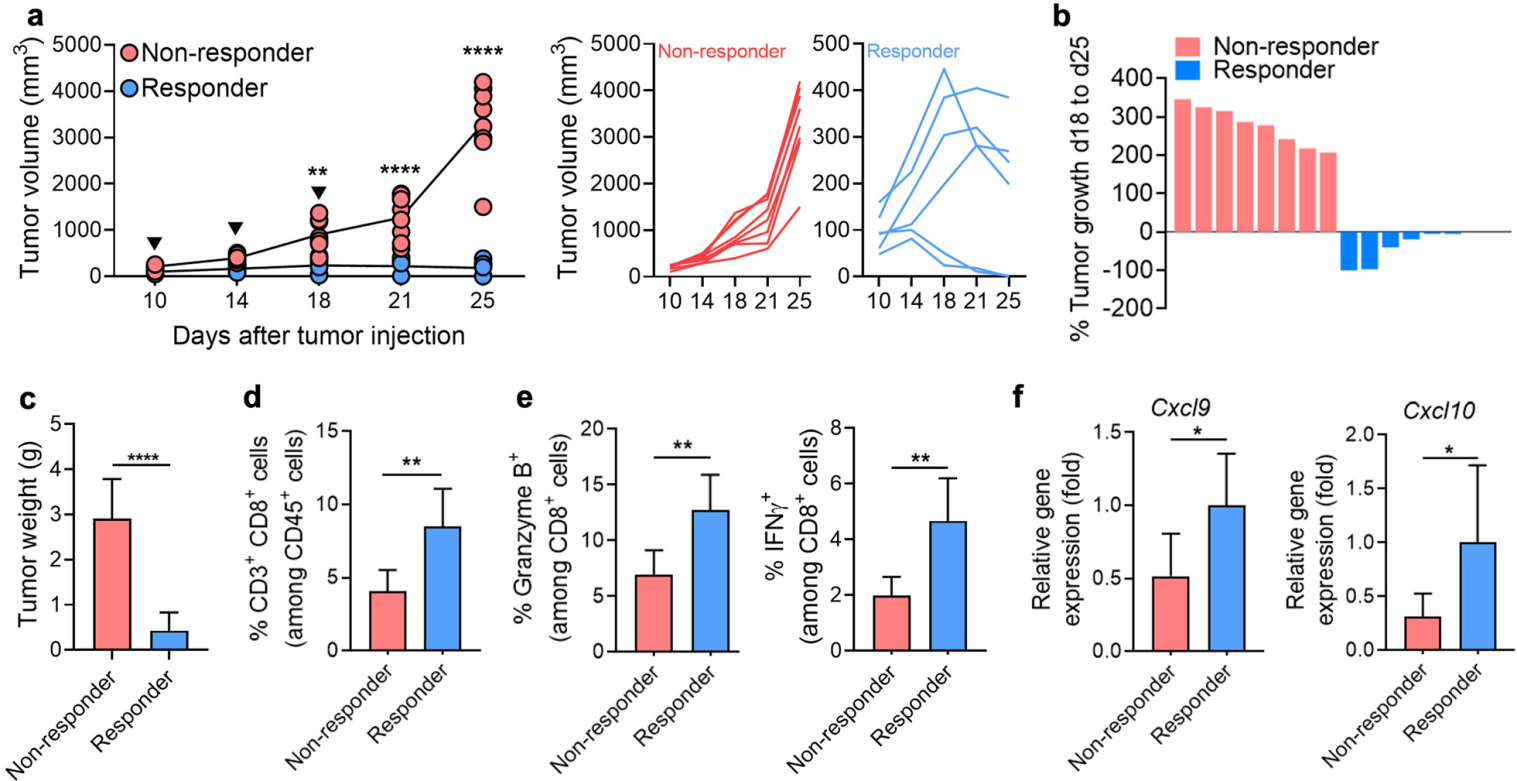
Increased frequency and activity of CD8^+^ T cells in responders to anti-PD1 treatment. B16F10-Luc tumor-bearing mice were treated with 100 μg of anti-PD1 (black filled inverted triangle) on day 10, day 14, and day 18. Tumors were obtained 15 d after initiating the anti-PD1 treatment. **a** Average (left) and individual (right) tumor volume following anti-PD1 treatment in B16F10-Luc tumor-bearing mice (non-responders, n = 8; responders, n = 6). **b** Response assessed by % of tumor growth between d 18 and d 25 post-tumor inoculation. (non-responders, n = 8; responders, n = 6). **c** Tumor weight after anti-PD1 treatment. (non-responders, n = 8; responders, n = 6). **d** Frequency of cytotoxic CD8^+^ T cells (gated on the CD45^+^ CD3^+^ CD8^+^ population) (n = 6/group). **e** Population percentages of Granzyme B^+^ and IFNγ^+^ in tumor-infiltrating CD8^+^ T cells as determined by flow cytometry (n = 6/group). **f** Relative mRNA expression of *Cxcl9* and *Cxcl10* in tumors of non-responders and responders (n = 6/group). Data are presented as mean ± SEM. Statistical significance was determined using a two-tailed Student’s *t*-test. Data shown are representative of three independent experiments performed. *p < 0.05; **p < 0.01; ****p < 0.0001

Effective anti-PD1 therapies induce the reactivation of immune cells to inhibit tumor growth. To investigate the effect of anti-PD1 in the immune cell compartment, we established an experimental model that randomly responds to anti-PD1 treatment. After inoculation with B16F10-Luc melanoma cells (3 × 10^5^, s.c., upper left and right flank), mice were treated with 100 μg of anti-PD1 three times every 4 d. We first confirm that the tumor size and weight were reduced in the responder group (Fig. 2a, c). We compared the tumor volume at d 18 baseline lesion volumes with d 28 post-therapy lesion volumes (Fig. 2b) [31].

In this setting, the ratio of responders to non-responders was approximately 4:6. As anti-PD1 directly targets T cells, we also observed T cell activation upon anti-PD1 treatment. As expected, infiltration of cytotoxic CD8^+^ T cells increased in the responder group (Fig. 2d). Moreover, their anti-tumor activity was further confirmed by the presence of granzyme B^+^ and IFNγ^+^ staining in CD8^+^ T cells from responders (Fig. 2e). To understand why T cell infiltration is increased, we examined the expression of *Cxcl9* and *Cxcl10*, which are important chemoattractants of T cells [32]. *Cxcl9* and *Cxcl10* expression was higher in responder than in non-responder tumors (Fig. 2f).

### Responders to anti-PD1 treatment show increased tumor-associated macrophages

**Fig. 3:**
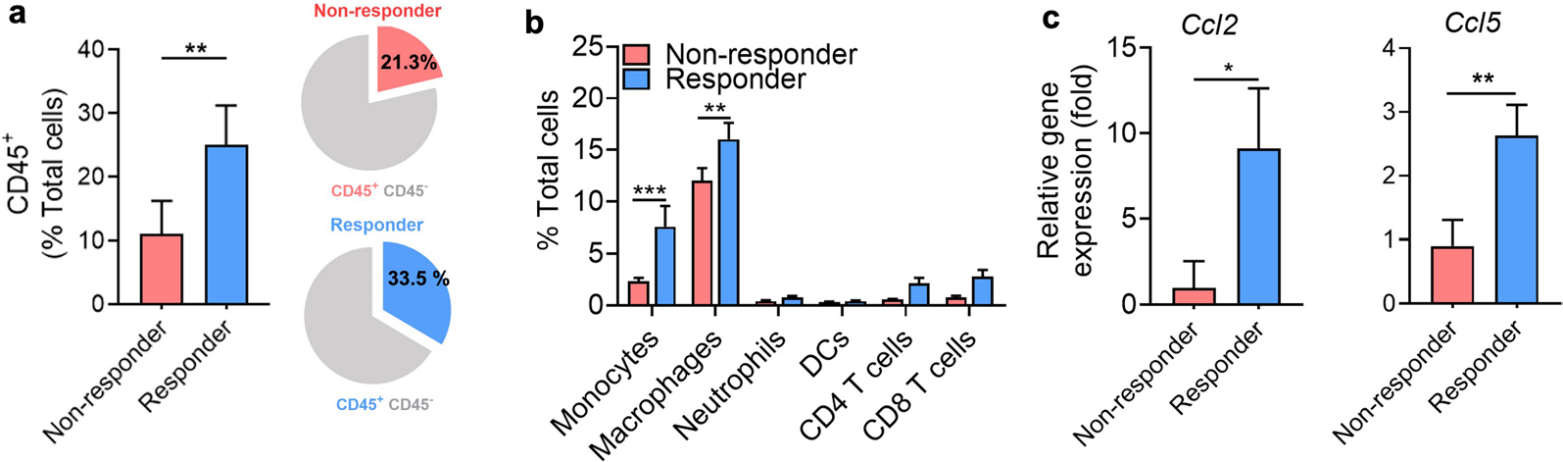
Augmented frequency of tumor-associated macrophage in responders to anti-PD1 treatment. **a** Percentage of CD45^+^ and CD45^−^ cells in non-responder and responder tumors at day 15 after anti-PD1 treatment (non-responders, n = 6; responders, n = 5). **b** The frequency of major immune cell types in the tumor microenvironment was measured by flow cytometry (non-responders, n = 6; responders, n = 5). **c** Relative mRNA expression of *Ccl2* and *Ccl5* in tumors of non-responders and responders (n = 3/group). Data are presented as mean ± SEM. Statistical significance was determined using a two-tailed Student’s *t*-test, and ANOVA with Tukey’s post-test was performed for multiple comparisons. Data shown are representative of three independent experiments performed. *p < 0.05; **p < 0.01; ***p < 0.001

Next, we investigated the differences in myeloid cells infiltrating the tumors between non-responders and responders. Consistent with previous studies, infiltration of CD45^+^ cells increased in response to anti-PD1 treatment (Fig. 3a). Further detailed analyses of infiltrated CD45^+^ cells revealed that, aside from lymphoid cells, including CD8^+^ T cells, the most significant increase was observed in TAMs (Fig. 3b, Additional file 1: Fig. S1). RT-PCR analysis showed a shift in chemokines related to macrophage recruitment, including *Ccl2* and *Ccl5* (Fig. 3c), suggesting the enhanced recruitment of macrophages. Thus, we aimed at the non-invasive imaging of TAMs that could provide information on immune cell changes in the TME during anti-PD1 treatment, which is associated with treatment responsiveness.

### [^68^ Ga]Ga-MSA-PET imaging shows different tumor-associated macrophage states in response to anti-PD1 treatment

**Fig. 4:**
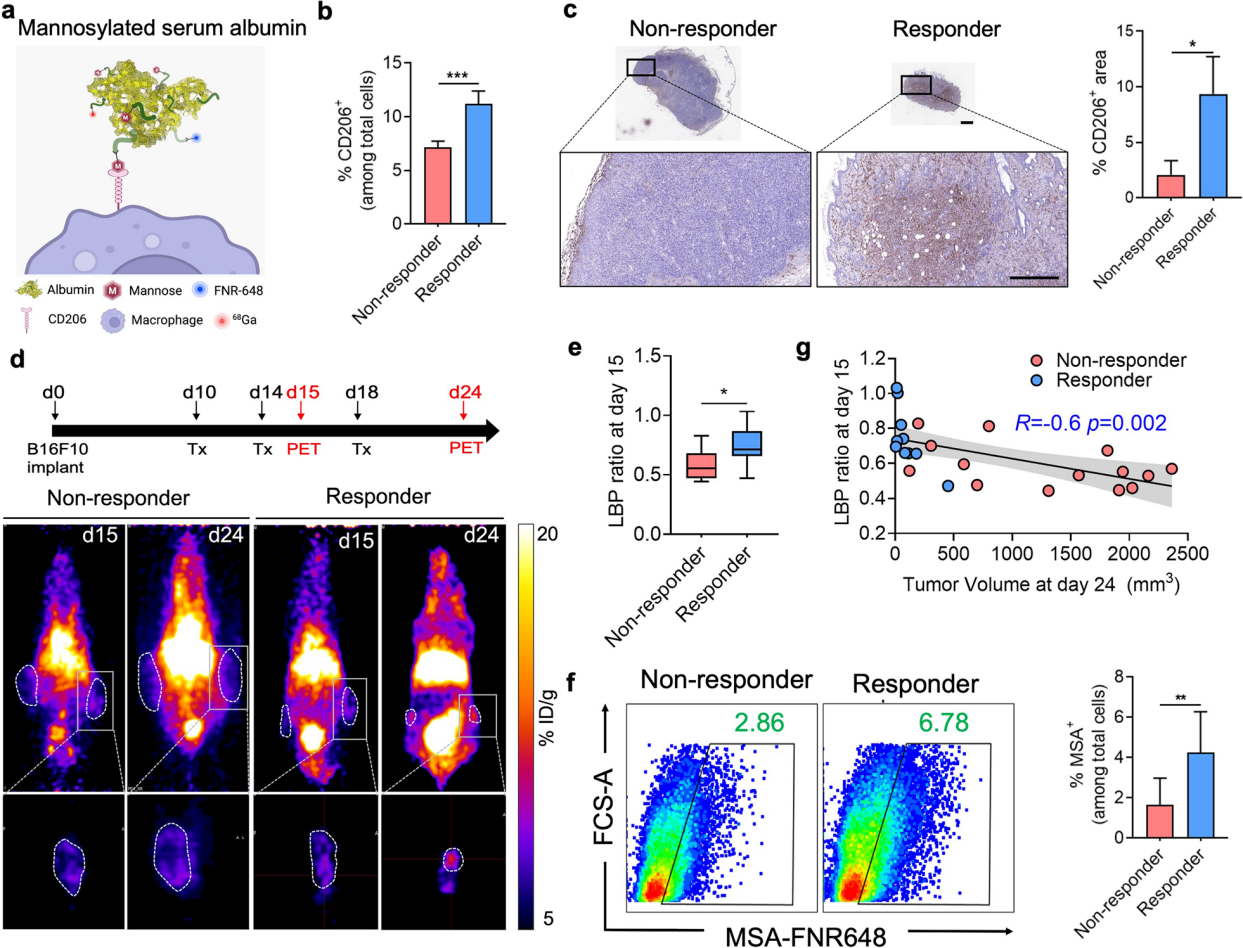
Different Imaging patterns of [^68^Ga]Ga-MSA nanoparticle in responders and non-responders to anti-PD1 treatment. **a** Schematic illustration of MSA. **b** Flow cytometry for the frequency of CD206^+^ cells in non-responder and responder tumors (n = 4/group). **c** Quantification of CD206 immunohistochemistry-stained tumor tissues from non-responders and responders. **d** Overview of experimental representative PET images of B16F10-Luc tumor-bearing mice according to the response to anti-PD1 treatment. Panels below the coronal whole-body PET images are cropped sagittal sections of the tumors. The scale bar of the image was adjusted for the image of the tumor site. **e** Measurement of Lesion to Blood pool (LBP) ratio at day 15 in the anti-PD1 treated tumor (non-responders, n = 14; responders, n = 10). **f** Representative flow cytometric dot plot (left) and percentages of MSA^+^ cells as determined by flow cytometry (right) (non-responders, n = 9; responders, n = 7). **g** [^68^Ga]Ga-MSA uptake measured by LBP at the early phase of anti-PD1 treatment (day 15) was negatively correlated with the tumor volume at late phase (day 24) (non-responders, n = 14, responders n = 10). Scale bar = 1 mm for upper images and 300 μm for magnified images. Data are presented as mean ± SEM. Statistical significance was determined using the two-tailed Student’s *t*-test. *p < 0.05; **p < 0.01; ***p < 0.001.

We developed albumin-based nanoplatform, MSA, for targeting the mannose receptor (CD206) highly expressed in TAMs (Fig. 4a) [25–27]. Interestingly, tumors treated with anti-PD1 showed significantly increased CD206 expression on TAMs, regardless of reactivity. Only 65% of TAMs expressed CD206 in the isotype control group, but most of the TAMs in the anti-PD1 treatment group expressed CD206 (Additional file 2: Fig. S2a–d). We also observed a significant increase in the CD206-expressing cell population in the tumor through flow cytometry (Fig. 4b). Additionally, quantifying CD206 stained tumor tissue revealed that the number of CD206^+^ cells increased at the tumor core in the responder compared to the non-responder (Fig. 4c). Next, we investigated whether [^68^Ga]Ga-MSA nanoparticle could non-invasively detect the infiltration of CD206-expressing TAMs according to the response to anti-PD1. Two hours after the administration of [^68^Ga]Ga-MSA nanoparticle on days 15 and 24, we conducted PET imaging. Furthermore, we examined the location of these immune cells in the tumor. Interestingly, we observed that the accumulation of [^68^Ga]Ga-MSA nanoparticle evenly throughout the tumor in responders but only at the tumor margin in the non-responders (Fig. 4d). Upon quantitative measurement, the maximum LBP ratio of the responders was significantly higher than that of non-responders (Fig. 4e). In line with [^68^Ga]Ga-MSA nanoparticle imaging, further analysis confirmed increased MSA uptake in the responder tumors, albeit with a very small size (Fig. 4f). We thus found that tumor size and the degree of [^68^Ga] Ga-MSA nanoparticle uptake showed a negative correlation (Fig. 4g).

**Fig. 5:**
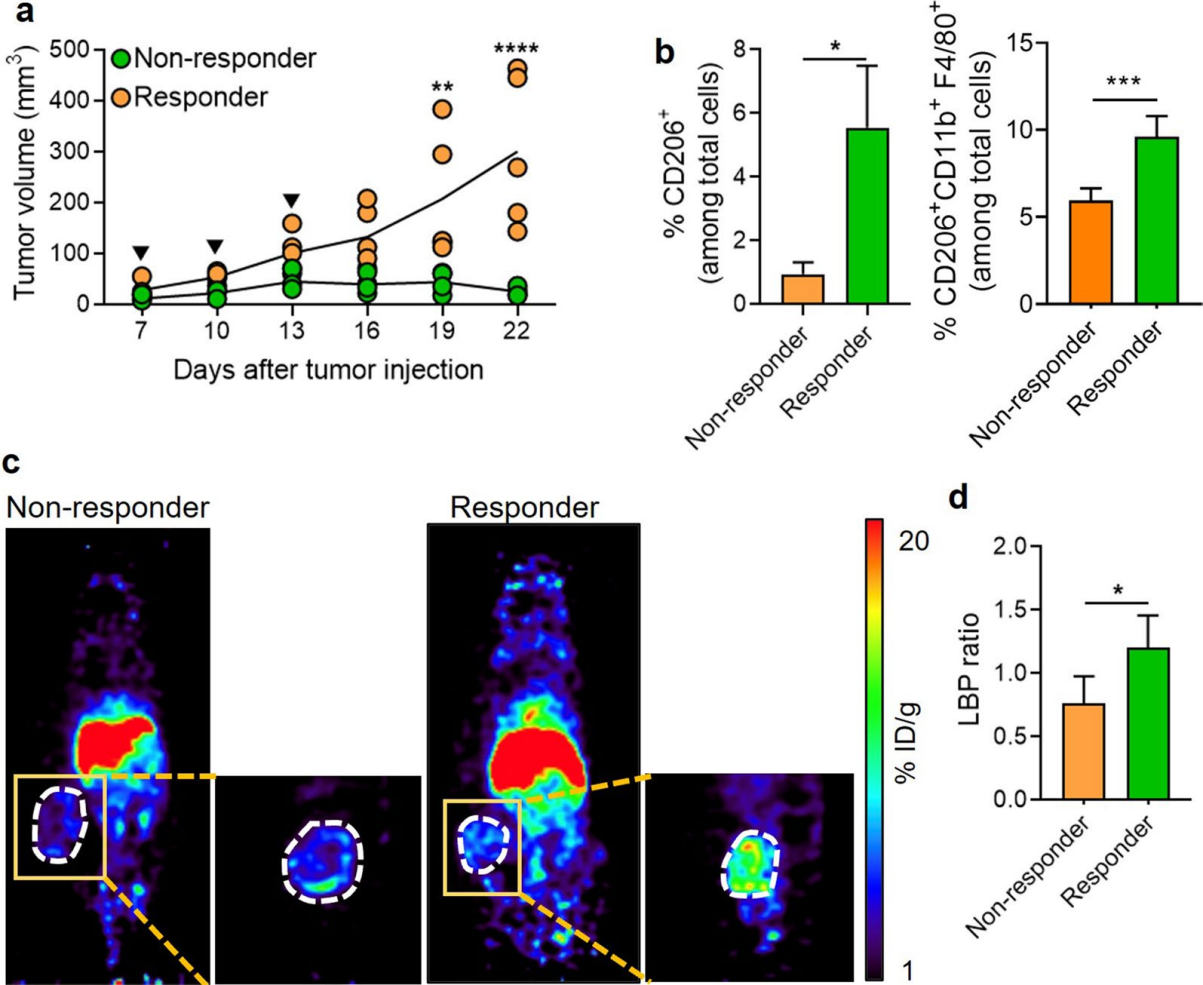
Different Imaging patterns of [^68^Ga]Ga-MSA nanoparticle in MC38-Luc tumor-bearing mouse model after anti-PD1 treatment. MC38-Luc tumor-bearing mice were treated with 100 μg of anti-PD1 (black filled inverted triangle) on day 7, day 10, and day 13. Experiments were conducted 15 d after initiating the anti-PD1 treatment. **a** MC38-Luc tumor volumes in mice treated with anti-PD1 (non-responders, n = 5; responders, n = 4). **b** Flow cytometry for the frequency of CD206^+^ cells in non-responder and responder tumors (non-responders, n = 5; responders, n = 4). **c** Overview of experimental representative PET images of MC38-Luc tumor-bearing mice that responded or failed to respond to anti-PD1 treatment. **d** Measurement of LBP ratio at day 22 in the anti-PD1 treated tumor (non-responders, n = 5; responders, n = 4). Similar results were obtained from two independent experiments. *p < 0.05; **p < 0.01; ***p < 0.001; ****p < 0.0001

Further imaging study was conducted in MC38-Luc tumor-bearing mice to investigate if the same phenomenon could be observed with [^68^Ga]Ga-MSA nanoparticle imaging in tumors other than melanoma. Consistent with the anti-PD1-response of B16F10, higher frequencies of CD206^+^ cells were observed in responders of MC38-Luc models (Fig. 5a, b); furthermore, the accumulation of [^68^Ga]Ga-MSA nanoparticle of the responders was higher than non-responders and appeared throughout the tumor (Fig. 5c, d). Collectively, these findings demonstrate a significant difference in the frequency and location of TAMs within the TME.

### The tumor microenvironment altered by anti-PD1 treatment allows macrophages to induce infiltration and activation of CD8^+^ T cells

**Fig. 6:**
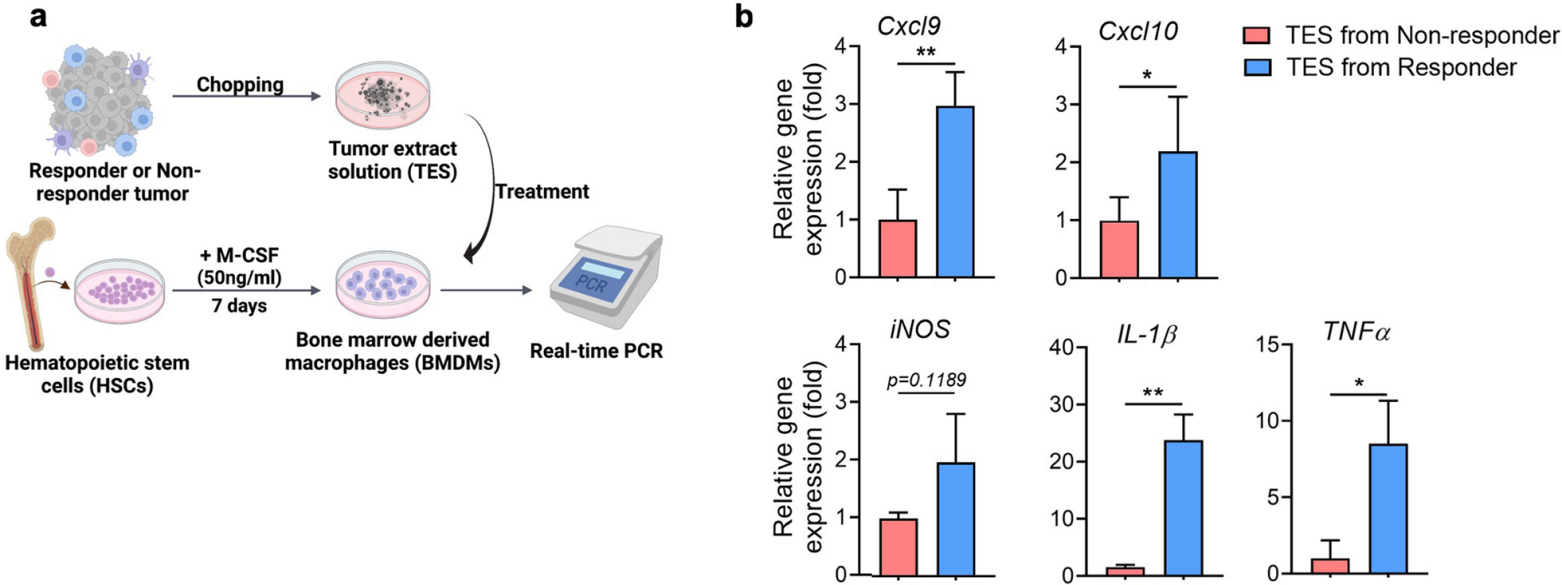
Tumor explant supernatant induces functional remodeling of the macrophages. **a** Schematic overview of the experimental design. **b** Relative mRNA expression of *Cxcl9, Cxcl10, iNos, IL-1β,* and *TNFα* in bone marrow-derived macrophages (BMDMs) treated with tumor explant supernatant (TES) obtained from tumors of non-responders or responders for 24 h (n = 3/group). Data are presented as mean ± SEM. Statistical significance was determined using the two-tailed Student’s *t*-test. Data shown are representative of three independent experiments conducted in triplicate. *p < 0.05; **p < 0.01

Given the well-described roles of TAMs in inducing T cell activation [33–35], we investigated the effect of ICI-induced TME alteration on TAMs, which in turn affected T cells. To this end, we prepared tumor explant supernatant (TES) from both responders and non-responders. Further, bone marrow-derived macrophages were treated with TES for 24 h, and *Cxcl9, Cxcl10, iNos, IL-1β, and TNFα* mRNA expression was determined. A simplified schematic of the experiment is shown in Fig. 6a. As expected, the expression of *Cxcl9* and *Cxcl10* was induced in macrophages exposed to responder-derived TES. Similarly, *iNos, IL-1b, and TNFα* expression was increased in responder-derived TES-treated macrophages, indicating the enhanced anti-tumor activity of macrophages for inducing T cell immunity (Fig. 6b). Collectively, our observations suggested that increased infiltration of TAMs identified by MSA nanoparticles could reflect TAM-mediated T-cell activation upon anti-PD1 treatment.

## Discussion

In this study, we investigated the capability of non-invasive imaging of TAMs using MSA nanoparticles to evaluate responsiveness to anti-PD1 in animal models. Previous studies have shown how ICI therapy affects the dynamics of intratumoral CD11b^+^ myeloid cells; this population comprises various immune cell types, including myeloid-derived suppressor cells (MDSCs), neutrophils, and natural killer cells (NK cells) [36]. As MDSCs represent an immunosuppressive phenotype and NK cells possess cytotoxic abilities similar to CD8^+^ T cells, it is difficult to discern responders from non-responders to ICI treatment by monitoring the entire CD11b^+^ population [37]. Nonetheless, overall immune cell infiltration in the tumor microenvironment is a predictive factor for therapeutic efficacy [38, 39]. Furthermore, our results showed increased overall immune cells in the TME after anti-PD1 treatment and the most frequent cell type was TAMs. Therefore, TAMs could be used as a surrogate imaging target to assess immune enrichment status of tumors. As a result, we showed the first non-invasive observation of dynamic changes in TAMs using MSA nanoparticles in response to ICI therapy.

To investigate whether overall macrophage enrichment could predict responsiveness to ICIs, we used MSA to monitor macrophages by targeting the mannose receptor (CD206) after anti-PD1 treatment. CD206 has generally known as the M2 marker [40–42]. However, increasing evidence suggests that this dualistic classification does not address the complex heterogeneity of macrophages, emphasizing the importance of unbiasedly describing the functions instead of the putative M1/M2 phenotypes [43, 44]. From this viewpoint, targeting CD206^+^ macrophages was interpreted as TAMs in our experiment. Further, the expression of MHCII and iNOS, which induce T cell activation, was increased in TAMs from responders, regardless of CD206 expression (Additional file 2: Fig. S2 E and F). In summary, the expression of CD206 cannot explain the functional state of TAMs; however, we only assessed the progression of anti-tumor responses to anti-PD-1 treatment through MSA imaging.

Monitoring TAM enrichment using non-invasive imaging could be used as a biomarker to evaluate the dynamic immune status of tumors. As immune status changes dynamically according to tumor progression and treatment, monitoring the tumor immune microenvironment could be important for the use of immune-oncology drugs. Conventional imaging, including CT and fluorodeoxyglucose PET, cannot differentiate between cancer and immune cells in the tumor microenvironment. Further, increased immune cells are a good predictive dynamic biomarker considering the mechanism of action of immuno-oncology drugs. Therefore, direct monitoring of immune cells using non-invasive imaging is promising for evaluating the responsiveness of ICIs in the early phase. These novel types of imaging methods, including [^68^Ga]Ga-MSA nanoparticle, could provide dynamic biomarkers reflecting the mechanisms of immune-oncology drugs.

TAMs exhibit distinct characteristics depending on their localization within the tumor core [45, 46]. Peripheral TAMs showed a more mature suppressive phenotype than those in the tumor core. Although the functional characteristics of TAMs involved in response to anti-PD1 remain to be elucidated, our finding implied that TAMs moved to the tumor core in the responder group after anti-PD1 treatment. This finding suggested the importance of spatial patterns of immune cells in TME in accordance with the response to treatment. As an example, tumors with pre-existing and homogeneously distributed CD8^+^ T cells at diagnosis are more likely to respond to ICI treatment, whereas tumors with a heterogeneous CD8^+^ T cell distribution show a weaker response [6]. This spatial pattern could be assessed by MSA nanoparticle imaging considering it provides spatial distribution of TAM infiltration, even though further tissue-level imaging study is needed. More specifically, as shown in previous research, TAMs induce impeding of T cells to inhibit the response to anti-PD1, and the results of TAM localization can be interpreted as induction of an immune response to stimulate infiltration of T cells after ICI treatment [16].

Overall, developing improved methods to noninvasively monitor therapeutic responses can provide individualized strategy for immune-oncology treatment. Because of the spatial and temporal heterogeneity of tumor microenvironment, biomarkers based on tissue samples have limitations in the representativeness of whole metastatic tumors [47]. For example, PD-L1 expression measured by immunohistochemistry, the most commonly used biomarker for immune checkpoint inhibitors, is commonly assessed by archive tissue samples for patients with metastatic tumors [48]. The temporal changes of tumor immune status mediated by several treatment before the immune-oncologic drugs is hard to be reflected by this archived samples. Therefore, non-invasive monitoring of tumor immune microenvironment is needed to evaluate temporal and spatial heterogeneity according to the treatment. Our approach aiming at the most frequent immune cell types in the TME, MSA imaging targeting TAMs, showed feasibility in monitoring the early change in immune cells during anti-PD1 treatment. Considering that 68 Ga-labeling for PET imaging and albumin-based nanoparticles are already clinically used, our method has the potential to be rapidly translated into the clinic.

## Conclusion

This study presents a non-invasive imaging method using MSA nanoparticles for monitoring TAMs as a factor in confirming the change of immune cells after ICI treatment. Monitoring of tumor immune status through imaging is increasingly necessary for the clinical use of immune checkpoint inhibitor therapy because of the heterogeneity of TME. Therefore, our approach that uses MSA nanoparticles for imaging TAMs as representative cell types in enriched immune cells according to the response to immune checkpoint inhibitors can be an imaging-based biomarker for precision oncology.

## Supplementary Information

**Additional file 1: Fig 1. **The gating strategy used to identify the different immune cell population. Immune cell populations were first gated based on the FSC-H and FSC-A, and SSA-H and SSC-A for single cell. After then, FSC-A and SSC-A positive portion were further gated based on CD45 expression. The subpopulations of the myeloid cells (**a**) and T cells (**b**) were gated based on specific surface markers as indicated in the panel.

**Additional file 2: Fig 2.** Anti-PD1 treatment polarizes tumor-associated macrophages toward a pro-inflammatory phenotype in responders (**a, c**) B16F10-Luc tumor-bearing mice were treated with 200 μg of anti-PD1 (black filled inverted triangle) on day 7, day 10, day 13, and day 16. Tumors were obtained 12 d after initiating the anti-PD1 treatment (n = 5/group). **b, d–f** B16F10-Luc tumor-bearing mice were treated with 100 μg of anti-PD1 (black filled inverted triangle) on day 10, day 14, and day 18. Tumors were obtained 15 d after initiating the anti-PD-1 treatment (non-responders, n = 6; responders, n = 4). **a, b** Average tumor volume in B16F10-Luc tumor-bearing mice treated with anti-PD1. **c, d** Frequency of CD206^+^ tumor-associated macrophage subsets after anti-PD1 treatment. (Gated on the CD45^+^ CD11b^+^ F4/80^+^ population). **e** Flow cytometry for the frequency of MHCII^+^ macrophages in non-responder and responder tumors (gated on the CD45^+^ CD11b^+^ F4/80^+^ population). **f** Flow cytometry for the frequency of iNOS^+^ macrophages in non-responder and responder tumors (gated on the CD45^+^ CD11b^+^ F4/80^+^ population). Data are presented as mean ± SEM. Statistical significance was determined using a two-tailed Student’s *t*-test. Data shown are representative of three independent experiments performed. *p < 0.05; **p < 0.01; ****p < 0.0001. *n.s* non-significant.

**Additional file 3: Table S1.** Primer sequences.

## Data Availability

The datasets generated during and/or analyzed during the current study are available from the corresponding author on reasonable request.

## Abbreviations

PD1: Programmed cell death protein 1
PD-L1: Programmed death-ligand 1
ICIs: Immune checkpoint inhibitors
TME: Tumor microenvironment
TAMs: Tumor-associated macrophages
PET: Positron emission tomography
MSA: Mannosylated- serum albumin
WT: Wild-type
FBS: Fetal bovine serum
PS: Penicillin/streptomycin
LBP: Lesion-to-blood pool
TES: Tumor explant supernatant

## References

[1] Sharma P, Allison JP (2015). The future of immune checkpoint therapy. Science.

[2] Sharma P, Hu-Lieskovan S, Wargo JA, Ribas A (2017). Primary, adaptive, and acquired resistance to cancer immunotherapy. Cell.

[3] Wolchok JD, Hoos A, O'Day S, Weber JS, Hamid O, Lebbé C, Maio M, Binder M, Bohnsack O, Nichol G (2009). Guidelines for the evaluation of immune therapy activity in solid tumors: immune-related response criteria. Clin Cancer Res.

[4] Borcoman E, Nandikolla A, Long G, Goel S, Le Tourneau C (2018). patterns of response and progression to immunotherapy. Am Soc Clin Oncol Educ Book.

[5] Guillerey C, Huntington ND, Smyth MJ (2016). Targeting natural killer cells in cancer immunotherapy. Nat Immunol.

[6] Waldman AD, Fritz JM, Lenardo MJ (2020). A guide to cancer immunotherapy: from T cell basic science to clinical practice. Nat Rev Immunol.

[7] Jiang X, Dudzinski S, Beckermann KE, Young K, McKinley E, JM O, Rathmell JC, Xu J, Gore JC (2020). MRI of tumor T cell infiltration in response to checkpoint inhibitor therapy. J Immunother Cancer.

[8] LaSalle T, Austin EE, Rigney G, Wehrenberg-Klee E, Nesti S, Larimer B, Mahmood U (2020). Granzyme B PET imaging of immune-mediated tumor killing as a tool for understanding immunotherapy response. J Immunother Cancer.

[9] Kristensen LK, Fröhlich C, Christensen C, Melander MC, Poulsen TT, Galler GR, Lantto J, Horak ID, Kragh M, Nielsen CH, Kjaer A (2019). CD4(+) and CD8a(+) PET imaging predicts response to novel PD-1 checkpoint inhibitor: studies of Sym021 in syngeneic mouse cancer models. Theranostics.

[10] Tavaré R, Escuin-Ordinas H, Mok S, McCracken MN, Zettlitz KA, Salazar FB, Witte ON, Ribas A, Wu AM (2016). An Effective immuno-PET imaging method to monitor cd8-dependent responses to immunotherapy. Cancer Res.

[11] Larimer BM, Wehrenberg-Klee E, Dubois F, Mehta A, Kalomeris T, Flaherty K, Boland G, Mahmood U (2017). Granzyme B PET imaging as a predictive biomarker of immunotherapy response. Cancer Res.

[12] Gibson HM, McKnight BN, Malysa A, Dyson G, Wiesend WN, McCarthy CE, Reyes J, Wei WZ, Viola-Villegas NT (2018). IFNγ PET imaging as a predictive tool for monitoring response to tumor immunotherapy. Cancer Res.

[13] Edwards KJ, Chang B, Babazada H, Lohith K, Park DH, Farwell MD, Sellmyer MA (2022). Using CD69 PET imaging to monitor immunotherapy-induced immune activation. Cancer Immunol Res..

[14] Zhao H, Wang C, Yang Y, Sun Y, Wei W, Wang C, Wan L, Zhu C, Li L, Huang G, Liu J (2021). ImmunoPET imaging of human CD8(+) T cells with novel (68)Ga-labeled nanobody companion diagnostic agents. J Nanobiotechnology.

[15] DeNardo DG, Ruffell B (2019). Macrophages as regulators of tumour immunity and immunotherapy. Nat Rev Immunol.

[16] Peranzoni E, Lemoine J, Vimeux L, Feuillet V, Barrin S, Kantari-Mimoun C, Bercovici N, Guerin M, Biton J, Ouakrim H (2018). Macrophages impede CD8 T cells from reaching tumor cells and limit the efficacy of anti-PD-1 treatment. Proc Natl Acad Sci U S A.

[17] Gubin MM, Esaulova E, Ward JP, Malkova ON, Runci D, Wong P, Noguchi T, Arthur CD, Meng W, Alspach E (2018). High-dimensional analysis delineates myeloid and lymphoid compartment remodeling during successful immune-checkpoint cancer therapy. Cell.

[18] Qu Y, Wen J, Thomas G, Yang W, Prior W, He W, Sundar P, Wang X, Potluri S, Salek-Ardakani S (2020). Baseline frequency of inflammatory Cxcl9-expressing tumor-associated macrophages predicts response to avelumab treatment. Cell Rep.

[19] Zhu Y, Knolhoff BL, Meyer MA, Nywening TM, West BL, Luo J, Wang-Gillam A, Goedegebuure SP, Linehan DC, DeNardo DG (2014). CSF1/CSF1R blockade reprograms tumor-infiltrating macrophages and improves response to T-cell checkpoint immunotherapy in pancreatic cancer models. Cancer Res.

[20] Sun J, Park C, Guenthner N, Gurley S, Zhang L, Lubben B, Adebayo O, Bash H, Chen Y, Maksimos M (2022). Tumor-associated macrophages in multiple myeloma: advances in biology and therapy. J Immunother Cancer.

[21] Ruffell B, Chang-Strachan D, Chan V, Rosenbusch A, Ho CM, Pryer N, Daniel D, Hwang ES, Rugo HS, Coussens LM (2014). Macrophage IL-10 blocks CD8+ T cell-dependent responses to chemotherapy by suppressing IL-12 expression in intratumoral dendritic cells. Cancer Cell.

[22] Molon B, Ugel S, Del Pozzo F, Soldani C, Zilio S, Avella D, De Palma A, Mauri P, Monegal A, Rescigno M (2011). Chemokine nitration prevents intratumoral infiltration of antigen-specific T cells. J Exp Med.

[23] Rodriguez PC, Quiceno DG, Zabaleta J, Ortiz B, Zea AH, Piazuelo MB, Delgado A, Correa P, Brayer J, Sotomayor EM (2004). Arginase I production in the tumor microenvironment by mature myeloid cells inhibits T-cell receptor expression and antigen-specific T-cell responses. Cancer Res.

[24] Gyori D, Lim EL, Grant FM, Spensberger D, Roychoudhuri R, Shuttleworth SJ, Okkenhaug K, Stephens LR, Hawkins PT (2018). Compensation between CSF1R+ macrophages and Foxp3+ Treg cells drives resistance to tumor immunotherapy. JCI Insight.

[25] Choi JY, Jeong JM, Yoo BC, Kim K, Kim Y, Yang BY, Lee YS, Lee DS, Chung JK, Lee MC (2011). Development of 68Ga-labeled mannosylated human serum albumin (MSA) as a lymph node imaging agent for positron emission tomography. Nucl Med Biol.

[26] Park JB, Suh M, Park JY, Park JK, Kim YI, Kim H, Cho YS, Kang H, Kim K, Choi JH (2020). Assessment of inflammation in pulmonary artery hypertension by (68)Ga-mannosylated human serum albumin. Am J Respir Crit Care Med.

[27] Lee S-P, Im H-J, Kang S, Chung S-J, Cho YS, Kang H, Park HS, Hwang D-W, Park J-B, Paeng J-C (2017). Noninvasive Imaging of myocardial inflammation in myocarditis using (68)Ga-tagged mannosylated human serum albumin positron emission tomography. Theranostics.

[28] Park JY, Song MG, Kim WH, Kim KW, Lodhi NA, Choi JY, Kim YJ, Kim JY, Chung H, Oh C (2019). Versatile and finely tuned albumin nanoplatform based on click chemistry. Theranostics.

[29] Park CR, Jo JH, Song MG, Park JY, Kim YH, Youn H, Paek SH, Chung JK, Jeong JM, Lee YS, Kang KW (2019). Secreted protein acidic and rich in cysteine mediates active targeting of human serum albumin in U87MG xenograft mouse models. Theranostics.

[30] Boktor RR, Walker G, Stacey R, Gledhill S, Pitman AG (2013). Reference range for intrapatient variability in blood-pool and liver SUV for 18F-FDG PET. J Nucl Med.

[31] Aide N, De Pontdeville M, Lopci E (2020). Evaluating response to immunotherapy with (18)F-FDG PET/CT: where do we stand?. Eur J Nucl Med Mol Imaging.

[32] Tokunaga R, Zhang W, Naseem M, Puccini A, Berger MD, Soni S, McSkane M, Baba H, Lenz HJ (2018). CXCL9, CXCL10, CXCL11/CXCR3 axis for immune activation—a target for novel cancer therapy. Cancer Treat Rev.

[33] House IG, Savas P, Lai J, Chen AXY, Oliver AJ, Teo ZL, Todd KL, Henderson MA, Giuffrida L, Petley EV (2020). Macrophage-derived CXCL9 and CXCL10 are required for antitumor immune responses following immune checkpoint blockade. Clin Cancer Res.

[34] Klug F, Prakash H, Huber PE, Seibel T, Bender N, Halama N, Pfirschke C, Voss RH, Timke C, Umansky L (2013). Low-dose irradiation programs macrophage differentiation to an iNOS(+)/M1 phenotype that orchestrates effective T cell immunotherapy. Cancer Cell.

[35] Pascual-García M, Bonfill-Teixidor E, Planas-Rigol E, Rubio-Perez C, Iurlaro R, Arias A, Cuartas I, Sala-Hojman A, Escudero L, Martínez-Ricarte F (2019). LIF regulates CXCL9 in tumor-associated macrophages and prevents CD8(+) T cell tumor-infiltration impairing anti-PD1 therapy. Nat Commun.

[36] Rashidian M, LaFleur MW, Verschoor VL, Dongre A, Zhang Y, Nguyen TH, Kolifrath S, Aref AR, Lau CJ, Paweletz CP (2019). Immuno-PET identifies the myeloid compartment as a key contributor to the outcome of the antitumor response under PD-1 blockade. Proc Natl Acad Sci USA.

[37] Theivanthiran B, Evans KS, DeVito NC, Plebanek M, Sturdivant M, Wachsmuth LP, Salama AK, Kang Y, Hsu D, Balko JM (2020). A tumor-intrinsic PD-L1/NLRP3 inflammasome signaling pathway drives resistance to anti-PD-1 immunotherapy. J Clin Invest.

[38] Bruni D, Angell HK, Galon J (2020). The immune contexture and Immunoscore in cancer prognosis and therapeutic efficacy. Nat Rev Cancer.

[39] Tavaré R, Danton M, Giurleo JT, Makonnen S, Hickey C, Arnold TC, Kelly MP, Fredriksson F, Bruestle K, Hermann A (2022). Immuno-PET monitoring of lymphocytes using the CD8-specific antibody REGN5054. Cancer Immunol Res.

[40] Murray PJ, Allen JE, Biswas SK, Fisher EA, Gilroy DW, Goerdt S, Gordon S, Hamilton JA, Ivashkiv LB, Lawrence T (2014). Macrophage activation and polarization: nomenclature and experimental guidelines. Immunity.

[41] Mosser DM, Edwards JP (2008). Exploring the full spectrum of macrophage activation. Nat Rev Immunol.

[42] Li Y, Wu H, Ji B, Qian W, Xia S, Wang L, Xu Y, Chen J, Yang L, Mao H (2020). Targeted imaging of cd206 expressing tumor-associated M2-like macrophages using mannose-conjugated antibiofouling magnetic iron oxide nanoparticles. ACS Appl Bio Mater.

[43] Zhou X, Liu Y, Hu M, Wang M, Liu X, Huang L (2021). Relaxin gene delivery modulates macrophages to resolve cancer fibrosis and synergizes with immune checkpoint blockade therapy. Sci Adv.

[44] Jaynes JM, Sable R, Ronzetti M, Bautista W, Knotts Z, Abisoye-Ogunniyan A, Li D, Calvo R, Dashnyam M, Singh A (2020). Mannose receptor (CD206) activation in tumor-associated macrophages enhances adaptive and innate antitumor immune responses. Sci Transl Med.

[45] Arlauckas SP, Garren SB, Garris CS, Kohler RH, Oh J, Pittet MJ, Weissleder R (2018). Arg1 expression defines immunosuppressive subsets of tumor-associated macrophages. Theranostics.

[46] Huang Y-K, Wang M, Sun Y, Di Costanzo N, Mitchell C, Achuthan A, Hamilton JA, Busuttil RA, Boussioutas A (2019). Macrophage spatial heterogeneity in gastric cancer defined by multiplex immunohistochemistry. Nat Commun.

[47] Sankar K, Ye JC, Li Z, Zheng L, Song W, Hu-Lieskovan S (2022). The role of biomarkers in personalized immunotherapy. Biomark Res.

[48] Wang DR, Wu XL, Sun YL (2022). Therapeutic targets and biomarkers of tumor immunotherapy: response versus non-response. Signal Transduct Target Ther.

