# Supplementary figures and images for: Mannosylated-serum albumin nanoparticle imaging to monitor tumor-associated macrophages under anti-PD1 treatment
Source: J Nanobiotechnology. 2023 Jan 27;21:31. doi: 10.1186/s12951-023-01791-9 (PMC9881286; doi:10.1186/s12951-023-01791-9)

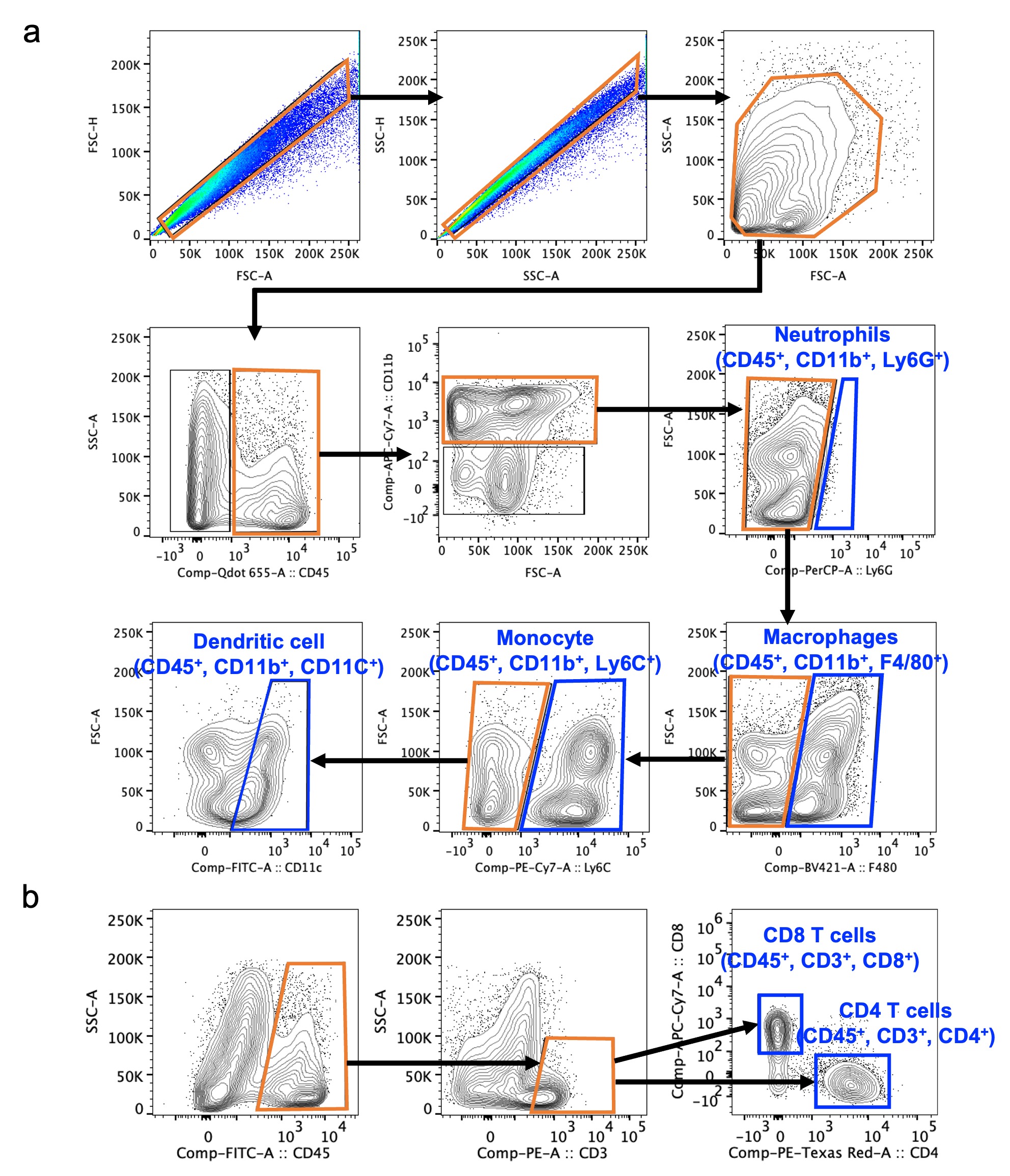

Supplement: Supplementary file 1 — Additional file 1: Fig 1. The gating strategy used to identify the different immune cell population. Immune cell populations were first gated based on the FSC-H and FSC-A, and SSA-H and SSC-A for single cell. After then, FSC-A and SSC-A positive portion were further gated based on CD45 expression. The subpopulations of the myeloid cells (a) and T cells (b) were gated based on specific surface markers as indicated in the panel. [file 12951_2023_1791_MOESM1_ESM.jpg]

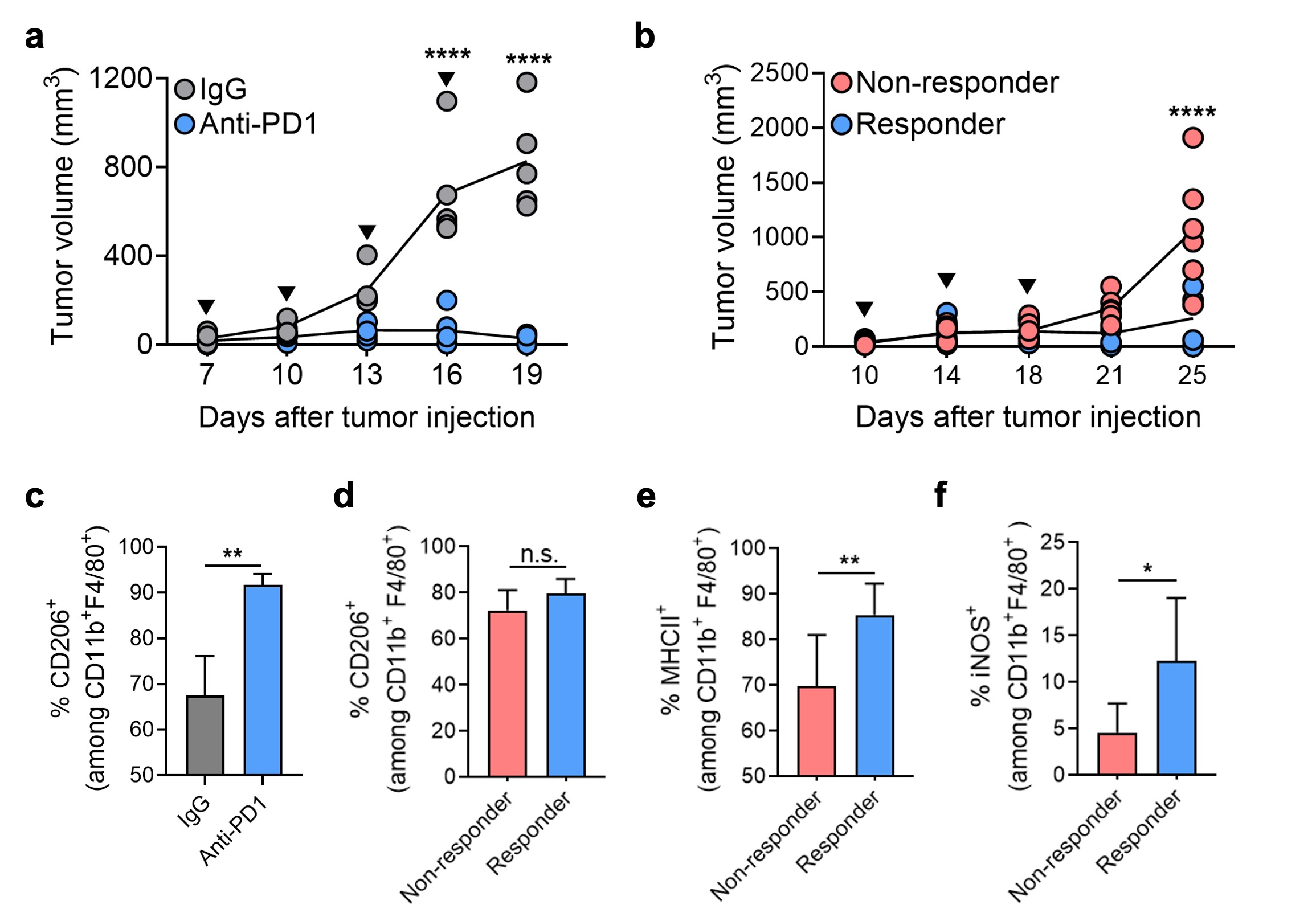

Supplement: Supplementary file 2 — Additional file 2: Fig 2. Anti-PD1 treatment polarizes tumor-associated macrophages toward a pro-inflammatory phenotype in responders (a, c) B16F10-Luc tumor-bearing mice were treated with 200 μg of anti-PD1 (black filled inverted triangle) on day 7, day 10, day 13, and day 16. Tumors were obtained 12 d after initiating the anti-PD1 treatment (n = 5/group). b, d–f B16F10-Luc tumor-bearing mice were treated with 100 μg of anti-PD1 (black filled inverted triangle) on day 10, day 14, and day 18. Tumors were obtained 15 d after initiating the anti-PD-1 treatment (non-responders, n = 6; responders, n = 4). a, b Average tumor volume in B16F10-Luc tumor-bearing mice treated with anti-PD1. c, d Frequency of CD206+ tumor-associated macrophage subsets after anti-PD1 treatment. (Gated on the CD45+ CD11b+ F4/80+ population). e Flow cytometry for the frequency of MHCII+ macrophages in non-responder and responder tumors (gated on the CD45+ CD11b+ F4/80+ population). f Flow cytometry for the frequency of iNOS+ macrophages in non-responder and responder tumors (gated on the CD45+ CD11b+ F4/80+ population). Data are presented as mean ± SEM. Statistical significance was determined using a two-tailed Student’s t-test. Data shown are representative of three independent experiments performed. *p < 0.05; **p < 0.01; ****p < 0.0001. n.s non-significant. [file 12951_2023_1791_MOESM2_ESM.jpg]
