# Supplementary material for: Mannosylated-serum albumin nanoparticle imaging to monitor tumor-associated macrophages under anti-PD1 treatment
Source: J Nanobiotechnology. 2023 Jan 27;21:31. doi: 10.1186/s12951-023-01791-9 (PMC9881286; doi:10.1186/s12951-023-01791-9)
Supplement: Supplementary file 3 — Additional file 3: Table S1. Primer sequences. [file 12951_2023_1791_MOESM3_ESM.docx]

Additional file 3: Table S1. Primer sequences

| *Ccl2* | F: 5′-CTC CAG CCT ACT CAT TGG GAT CA-3′  R: 5′-CTC CAG CCT ACT CAT TGG GAT CA-3′ |
| --- | --- |
| *Ccl5* | F: 5′-ACT CCC TGC TGC TTT GCC TAC-3′  R: 5′- ACT TGC TGG TGT AGA AAT ACT-3′ |
| *Cxcl9* | F: 5′-AGC CCC AAT TGC AAC AAA AC-3′  R: 5′-TCT TCA CAT TTG CCG AGT CC-3′ |
| *Cxcl10* | F: 5′-GGG CCA TAG GGA AGC TTG AA-3′  R: 5′-GGA TTC AGA CAT CTC TGC TCA TCA-3′ |
| *iNos* | F: 5′-TCC TGG AGG AAG TGG GCC GAA G -3′  R: 5′-CCT CCA CGG GCC CGG TAC TC-3′ |
| *Il-1β* | F: 5’-CTC AAT GGA CAG AAT ATC AAC CAA CA-3’  R: 5’-ACA GGA CAG GTA TAG ATT CTT TCC TTT G-3’ |
| *TNFα* | F: 5’-CACCCCGAAGTTCAGTAGACA-3’  R: 5’- GAACTGGCAGAAGAGGCACT-3’ |
| *18SrRNA* | F: 5′- GCA ATT ATT CCC CAT GAA CG -3′  R: 5′- GGC CTC ACT AAA CCA TCC AA -3′ |
